# Regulating Alcohol: Strategies Used by Actors to Influence COVID-19 Related Alcohol Bans in South Africa

**DOI:** 10.3390/ijerph182111494

**Published:** 2021-10-31

**Authors:** Yandisa Ngqangashe, Maddie Heenan, Melanie Pescud

**Affiliations:** 1 School of Regulation and Global Governance, The Australian National University, 8 Fellows Road, Canberra, ACT 2601, Australia; melanie.pescud@anu.edu.au; 2 ANU College of Asia and the Pacific, The Australian National University, 8 Fellows Road, Canberra, ACT 2601, Australia; 3 The Australian Prevention Partnership Centre, The Sax Institute, Glebe, NSW 2037, Australia; maddie.heenan@saxinstitute.org.au; 4 The George Institute for Global Health, Level 5/1 King St., Newtown, NSW 2042, Australia

**Keywords:** alcohol regulation, prohibition, COVID-19, industry, government

## Abstract

South Africa has used intermittent alcohol prohibitions and restrictions as a strategy to relieve hospitals of alcohol-related trauma cases and spare services for COVID-19 caseloads. Alcohol regulation is highly contested and involves a diverse range of actors who influence policies to align with their interests. This study sought to examine the strategies used by these actors to shape the COVID-19 related alcohol regulation in South Africa as presented by online news media. We found that the voice of pro-regulation actors is smaller and fragmented compared to opponents of the regulation as each actor seeks to advance their own interests. Despite the regulations initially being framed as a COVID-19 public health measure, pro-regulation government ministries, such as police and transport, perceive the regulations as a way of reducing existing (pre-pandemic) alcohol-related harm, such as crime, road-traffic injuries, and gender-based violence. The pre-existing failures in the alcohol regulatory environment and the current policy momentum created by COVID-19 could present an opportunity to retain components of the new laws and improve alcohol regulation in South Africa. However, there is a dominant and cohesive alcohol industry voice that strongly opposes the regulations, citing economic impacts, illicit trade and lack of evidence on the positive effects of the alcohol bans. Strategies employed by industry include lobbying, framing, and litigation. The regulations implemented under the guise of COVID-19 prevention have presented valuable lessons for alcohol regulation more generally. However, whether these regulations translate to sustainable policy changes will depend upon how and if the strong industry voice is countered.

## 1. Introduction

The COVID-19 pandemic has brought the urgency of alcohol control to the forefront of public policy debates in South Africa. A study that was published in 2018 reported that about 33% of the South African population were reported to consume alcohol, of which 43% reported binge drinking [1]. The role of alcohol in contributing to various major public health threats, such as non-communicable diseases, violence and personal injury, poor mental health, and infectious diseases are well known [2,3]. In South Africa, alcohol contributes to all facets of the country’s quadruple burden of disease–non-communicable disease, infectious disease, maternal and child health, and injury and trauma [4]. With regard to injury and trauma, South Africa has some of the highest rates of trauma-related mortality, at six times the global rate, as well as road traffic injuries, which were double the global average in 2007 [4]. In 2015, it was estimated that at least 62,300 people died from alcohol-attributable causes, a majority of which were from low socioeconomic groups [1]. Alcohol-related injuries account for a significant proportion of the trauma burden in South African Hospitals [5]. Knowing this, the South African government has used intermittent prohibition and restrictions of alcohol sales as a strategy to reduce the incidence of trauma cases presenting to hospitals, in attempts to preserve the capacity of the health system and cope with COVID-19 patient loads.

The problems associated with excessive alcohol use warrant crosscutting solutions to reduce its consumption. The regulation of the provision, supply, and acquisition of alcohol through policies is one of the potential strategies to reduce alcohol consumption [6]. Alcohol policies are already proving to be an effective strategy for reducing consumption and associated harm in high-income countries, but this is not the case for low to middle-income countries (LMICs) [3]. The lack of comprehensive alcohol regulation in LMICs has previously been attributed to a lack of political will, aggressive industry strategies to increase the uptake of alcohol consumption, and perceived benefits of the alcohol industry to economies and jobs [3,6]. While there have been efforts to regulate alcohol through labeling requirements and advertising restrictions, South Africa does not have a comprehensive alcohol policy due to various factors, including strong industry opposition [7,8]. The regulation of alcohol is complex and involves multiple actors, such as the alcohol industry, consumers, and government. Each of these actors has varying interests and uses different strategies to influence the alcohol regulatory environment in ways that align with these interests [9]. This study seeks to examine how these actors influenced the COVID-19 related alcohol bans.

**Figure 1:**
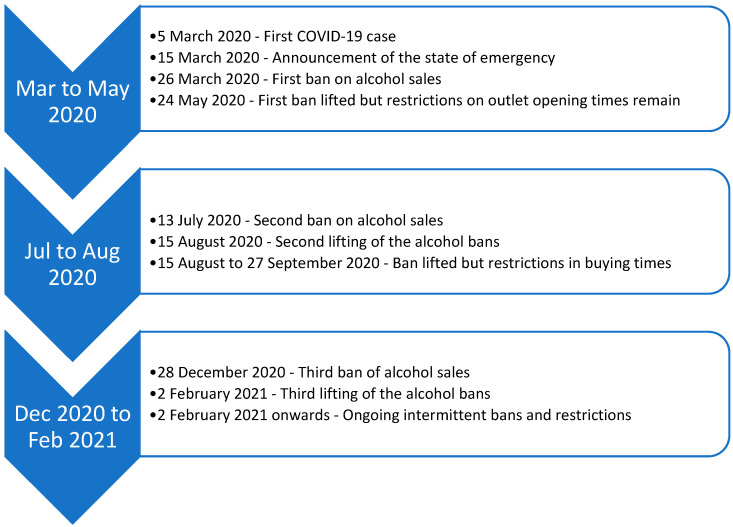
Timeline of events pertaining to the bans and trading hours restrictions of alcohol sales across the different levels of the COVID-19 lockdowns.

In March 2020, the South African government announced a state of national disaster and promulgated amendments to the Disaster Management Act of 2000 to provide a legislative framework specific to the prevention and management of the COVID-19 pandemic [10]. The Disaster Management Act provides a policy framework for disaster prevention, mitigation of severity, effective response, and post-disaster recovery in cases of national disaster. The regulations include restrictions and prohibitions of non-essential goods including alcohol [10]. The rationale for the restriction on alcohol was to reduce social gatherings and to relieve hospitals of alcohol-related trauma cases in order to spare more health services for COVID-19 related care. In addition, co-use of substances, such as tobacco and alcohol by the large proportion of vulnerable citizens with existing illnesses was seen as a potential risk factor for COVID-19 complications [11,12]. The regulations were loosened or tightened frequently in response to new COVID-19 case numbers. When the cases were increasing there was a complete prohibition of alcohol sales and when the cases were declining the prohibition was lifted but restrictions on days and times of sales remained. See Figure 1 for a timeline of events pertaining to the banning and relaxation of alcohol sales across the different levels of the COVID-19 lockdowns.

South Africa is a constitutional democracy with a three-sphere system of government consisting of national, provincial, and local governments. Each of these arms have the power to develop legislation applicable in their own areas. The legislative process usually involves multiple stages of consultation and iterations of draft bills before being signed off by the President. However, the COVID-19 regulations, including the alcohol regulations, were emergency responses implemented under the Disaster Management Act that unlike regulations implemented under the standard South African legislative process, did not require consultation. The country’s emergency response to COVID-19 is being coordinated by a National Command Council that is comprised of 20 national ministers and directors-general (permanent secretary) of 20 departments, chaired by the President and Ministry of Corporative Governance and Traditional Affairs.

The COVID-19 alcohol regulations were dynamic, easing and tightening frequently throughout the lockdown in response to the country’s burden of COVID-19 infections. News media played a significant role in the real-time dissemination of these regulations via news bulletins, live broadcasts of the Ministers’ press conferences, live tweets, and online news websites. News media also kept the nation informed on the impacts of these regulations and provided an outlet for different actors to express their concerns or support for the regulations. In doing so, news media, especially online news media that is easily accessible through social media and mobile phones, played a critical role in shaping the public discourse on COVID-19.

The media plays a critical role in keeping alcohol policy issues on the agenda [9]. Media may also craft opinions by giving space in the form of Op-Eds and letters to the editor, as observed in one study on the news media framing of the implementation of alcohol regulations in South Africa [9]. Multiple actors with varying interests in the alcohol policy landscape use different strategies, including the media, to influence policies, such as restrictions on alcohol sales and advertising, and implementation of product warning labels [8].

In light of the alcohol-related social and health harm in South Africa, the appetite for regulation is high [7,8]. This may imply that there will be further changes to the alcohol regulatory landscape. According to agenda-setting theories, the strategies used by actors to counter or support regulations will play a role as to whether or not changes materialize [13,14]. This study will explore the actors and types of strategies they employ to influence the alcohol regulatory environment during the South African COVID-19 alcohol restrictions, as reported by online news media. This will provide a snapshot of the relevant policy actors, as well as lessons for ensuring the political priority of alcohol regulations beyond COVID-19.

## 2. Materials and Methods

### 2.1. Analytical Framework

The power of actors involved in a policy issue and the strategies they employ to shape the policy environment, including how they frame problems, play a critical role in determining what issues gain political priority and what types of solutions are proposed [14,15]. The way actors frame and understand policy issues is not only influenced by the salience of the issue but by their values, belief systems, and culture [16,17]. In the highly contested policy-making space, various actors use framing to advance their agenda by defining the problem, attributing causality, assigning responsibility, and proposing or justifying solutions, such as policies or interventions [16,17]. Guided by this literature, we developed a coding framework to identify the key actors and the strategies they employed to shape COVID-19 related alcohol regulations in South Africa [13,14,18,19].

### 2.2. Study Design

Directed qualitative content analysis was used as the research methodology [20]. This entailed a deductive approach to coding and analysis whereby the research team sought to use and extend pre-existing theories to guide the analysis [20,21]. The study entailed the development of an analytical framework to guide the coding, extraction of news articles from news websites, piloting of the coding framework, and a content analysis of newspaper articles as per Hsieh & Shannon, 2005.

### 2.3. Data Collection

A total of 142 online news media articles were obtained from online news website archives of the South African Broadcasting Corporation (SABC) https://www.sabcnews.com/sabcnews/ accessed 28 February 2021 (N = 53 articles) and News24 https://www.news24.com/ accessed 28 February 2021 (N = 89). SABC news online is a digital news media platform for the public state-owned broadcaster—South African Broadcasting Corporation. As a national broadcaster, the SABC has a public interest mandate and hosts content from the most consumed free-to-air news bulletins in South Africa. News24 is an independent digital-only platform that has the highest reach in South Africa [20]. News24 captures multiple perspectives because the websites also host news content from newspapers and magazines that belong to the same parent company and are more inclined towards business interests [21]. Together these platforms are widely consumed, current, and capture perspectives of diverse actors.

The articles were obtained online from the archives of each website using the search terms “alcohol ban” and “alcohol COVID-19”. Our research team extracted and analyzed news media content published from 15 March 2021 (announcement of the state of emergency) to 7 February 2021 (one week after the third alcohol ban was lifted). We excluded articles that were about alcohol but unrelated to COVID-19. Opinion pieces were excluded except for those from key actors, such as government ministers and the alcohol industry. The article search was completed by the lead researcher (YN).

### 2.4. Data Analysis

All news articles were uploaded into QSR Nvivo12 (QSR International Pty Ltd., Australia) for coding and analysis. The coding framework consisted of three major deductive codes from the literature [11,15,16,17]: (1) the actors involved or presented in the news articles, (2) the arguments they made for or against the prohibitions, and (3) the solutions or policy alternatives they proposed.

The coding was conducted by YN and MP in an iterative manner. Initially, YN and MP coded 20 articles together to test the coding framework. To begin, content from the set of 20 articles was coded line by line or in larger chunks of text to facilitate the identification of key concepts. These were subsequently grouped together into categories within the coding framework. Next, YN and MP worked separately to code the remaining articles, meeting each week for three weeks to discuss disagreements and new codes that were emerging. Once coding was complete, all three researchers (YN, MP, and MH) met at the same time on two separate occasions via zoom video call to conduct the thematic analysis. Each article was read together with themes identified on a sentence-by-sentence basis. Themes were then discussed and synthesized drawing upon Shiffman & Smith’s determinants of political priority—actor power, ideas, political contexts and nature of the issue [14], with analytical notes written up during each video call.

## 3. Results

The reporting of the views by different actors varied between the two news platforms; the reporting on News24 was more alcohol industry-oriented while the reporting on SABC News was more government-oriented. A number of actors’ views were represented within the two dominant online news media platforms, these included the government, the alcohol industry, and non-government organizations (NGOs). Each of the actors used different arguments to shape the COVID-19 alcohol regulatory environment to align with their interests. The most dominant arguments were health and the economy. Furthermore, actors who were against the ban proposed policy alternatives to the prohibitions. Below we describe each of the key actors in concert with the arguments and policy alternatives they proposed.

### 3.1. Government

The government actors represented in the media were the national Ministry of Health, Ministry of Cooperative Governance and Traditional Affairs (COGTA), and Ministries of Transport, Finance, and Police. There were also provincial government actors, such as premiers, provincial ministries of health, and alcohol regulatory entities. Even though decisions were made by a National Coronavirus Command Council (NCCC) that includes about 20 ministries, and is communicated by the President and COGTA Ministry, the voices that were presented in the media were not the NCCC but singular ministries with each ministry justifying the alcohol ban from the perspective of their interest. For example, the Ministry of Health and other related health actors consistently justified the alcohol ban as a means to reduce trauma cases and relieve pressure from the health system, while the Ministries of Transport and Police spoke to the alcohol bans in relation to reducing road accidents and crime statistics, respectively.

*The Health Department has asked the council to consider the impact of alcohol-related trauma cases on the health system.*—**SABC** **News** **(11** **July** **2020)**

*Police Minister Bheki Cele (Minister of Police) has reaffirmed his assertion that the alcohol ban during the national lockdown is the reason for the decrease in crime rates, this time waving crime statistics as evidence*.—**News24** **(22** **April** **2020)**

Throughout the articles, the goal of the President, Ministry of Health and COGTA Ministry was to explain and justify the alcohol bans. This was primarily done through evidence of the impact of alcohol bans by quoting hospital trauma admission statistics. The alcohol bans and this justification were also supported by research councils and public health academics who provided evidence to demonstrate the effectiveness of the bans in reducing hospital admissions.

*“Health services in several parts of the country reported that the prohibition of alcohol sales had significantly reduced the number of trauma cases seen on our hospitals over the year period”*—The president quoted by **SABC** **News** **(12** **January** **2021)**

In justifying the alcohol ban, there was an acknowledgment of its economic impact; however, the only alternative put forward was responsible drinking. In this instance, the government was placing responsibility on individual consumers, and this position reiterates typical industry messaging when it comes to the regulation of alcohol [22].

*“We are acutely aware that these restrictions have negatively affected businesses and threatened jobs in the hospitality, tourism and related industries”*—The president quoted by **SABC** **News** **(2** **February** **2021)**

*But Ramaphosa warned citizens not to view the lifting of restrictions as an opportunity to abuse alcohol—“I want to call on all of us to drink responsibly so that we do not experience a spike in trauma cases or an increase in infections due to reckless behaviour”*—The president quoted on **SABC** **News** **(2** **February** **2021**)

The Ministry of Police and Ministry of Transport were often quoted in the media expressing the desire for long-term regulation of alcohol. This positions the COVID-19 alcohol ban as a policy window [12] and demonstrates the spillover effects of the regulations. While there was some alignment in terms of the necessity of the bans, there were also different vested interests concerning the alcohol ban amongst the government actors. The Ministry of Health and COGTA Ministry interests were related to health while the interests of the Ministries of Transport and Police were related to pre-existing alcohol-related harm, such as gender-based violence, crime, and road traffic accidents. The appetite for using alcohol bans to address existing problems also demonstrated how COVID-19 has exposed and exacerbated many problems.

*Mbalula (Minister of Transport) says he is lobbying government to introduce legislation that there should be zero percent alcohol in drivers’ blood when tested.*—**SABC** **News** **(24** **August** **2020)**

*“It has been proven even though we have not given empirical evidence that when we release the statistics during the Easter holidays this year, we can link that to the present in relation to the impact of alcohol and impact of reckless driving on our roads”*—Minister of Transport quoted by **SABC** **news** **online** **(25** **August** **2020)**

*Police Minister Bheki Cele has reaffirmed his assertion that the alcohol ban during the national lockdown is the reason for the decrease in crime rates, this time waving crime statistics as evidence*—**News24** **(22** **April** **2020)**

While not dominant in the news media we reviewed, there were government ministries that opposed the alcohol ban. For example, the Ministry of Finance and South African Revenue Services emerged in the conversation regarding the economic impact of the ban.

*The NCCC is also expected to receive push back from the Economic Cluster, led by Finance Minister Tito Mboweni, who has detailed lockdown’s devastating socio-economic impact*—**News24** **(1** **December** **2020)**

*Amanda Lotheringen, director of monitoring complaints at the Department of Trade and Industry, said the government had lost R7.8 billion in direct tax and R5.8 billion in excise tax due to the ban*—**News24** **(12** **January** **2021)**

Partisan politics played a significant role as to whether provincial governments supported the alcohol prohibitions. Some Premiers and provincial health ministries from provinces under the leadership of the African National Congress (ANC) (a center-left democratic party—that is also the ruling party nationally and in eight of South Africa’s nine provinces) were quoted as lobbying the national government to reinstate the alcohol bans whenever there were surges in case numbers.

*The Eastern Cape Health Department says the ban on alcohol sales will help to ease the burden on health facilities. The province has also noted shortages of oxygen at health facilities in the province*—**SABC** **News** **(14** **July** **2020)**

*The North West Health MEC Madoda Sambatha is calling for a ban on the sale of alcohol. The North West health authorities are concerned about the level of non-compliance to lockdown regulations in the province*—**SABC** **News** **(27** **December** **2020)**

In contrast, the Democratic Alliance (DA) led province of the Western Cape consistently reported being against the alcohol bans. The DA is a pro-business right-wing liberal political party that is the main opposition party to the national government and currently holds power in the Western Cape Province. The Western Cape Provincial Government used different strategies to lobby the national government to relax the alcohol bans. These included lobbying the President and various ministers through letters, doubting the evidence of the impacts of alcohol bans on hospital admissions, arguing the negative economic impacts of COVID-19, and threatening litigation. There was also the use of vulnerability framing whereby the Western Cape Provincial Government highlighted the impact of COVID-19 on small businesses and minimum wage farming communities.

*“For as long as the Western Cape can assure access to health facilities for all Covid-19 patients, all businesses should be allowed to open safely, following clear health guidelines designed to slow the spread of Covid-19,”*—Western Cape Provincial Government quoted by **News24** **(7** **August** **2020)**

### 3.2. The Alcohol Industry

The alcohol industry actors whose views emerged in the newspaper articles included multinational alcohol corporations, alcohol retailers, wineries, liquor trader associations, microbrewers, and farmers. The most dominant perspective was from the top four multinational corporations—South African Breweries (SAB—owned by Anheuser-Busch InBev), Heineken, Diageo, and Distell Group. All alcohol industry actors were against alcohol prohibition and employed various strategies to influence the government to not introduce or to lift alcohol bans. The alcohol industry views were predominantly presented on News24. The industry used multiple strategies to influence the regulations. These included—framing, lobbying, and court cases. In terms of framing, the alcohol industry framed arguments against the alcohol bans in ways that emphasized the impacts of the alcohol ban on the broader economy, sustainability of small businesses, and job losses rather than profits of individual companies. In presenting losses and investment cancellations, the industry also used large figures that were presented in billions to magnify the economic impact. These figures were repeated in many articles by different actors within the alcohol industry, while at the same time, health was rarely mentioned. With each of the three bans, the industry repeatedly announced they would halt capital development even though this point was not a new development, and thus, old news.

*“We lost 117,000 jobs downstream and upstream in the broader industry. We believe that 800 small business enterprises are … facing bankruptcy,”*—CEO of an alcohol industry multinational corporation

*“Irreparable damage has already been done with our sector already losing 15% of South African craft breweries and 68% of those still trading having a decidedly negative outlook for the sustainability of their businesses going forward,”*—Chairperson of an alcohol industry association quoted by **News24** **(1** **August** **2020)**

*“The alcohol industry has already lost 118,000 jobs and projections show that a nine-week ban now will cost another 84,000 livelihoods and R15.5 billion in GDP.*—CEO of an alcohol industry multinational corporation quoted by **News24** **(3** **August** **2020)**

*SAB has been hit hard by the ban and yesterday announced that it was halting a R2.5 billion expansion plan in Durban. The 120,000 people employed in the industry are at risk of losing their job due to the ban*—**News** **24** **(4** **August** **2020)**

*South African Breweries (SAB) announced on Friday that it has cancelled a further R2.5 billion of capital investment following the third blanket ban on alcohol sales. This brings the brewer’s cancelled capital expenditure in SA since alcohol sales bans were introduced in 2020 to a total of R5 billion*—**News24** **(15 January 2021)**

There was also profiling of small business owners who had been impacted by the alcohol ban. This served to highlight vulnerability by referring to the ownership economy, female ownership specifically, and small family-owned businesses.

*Nomasonto is one of the 192,000 people who rely on taverns for their livelihood in South Africa, and without another source of income she was forced to join the many unemployed people in South Africa due to the alcohol ban*—**News24** **(25** **August** **2020)**

*“Although we are a small and independent business, we directly employ eight staff who in return are responsible for a total of 28 other people—children, spouses, parents. Even though we can’t sell any whisky, we still have rent due and salaries to pay so our staff can have roofs over their heads and food on their tables. We are literally having to borrow money from the bank to make ends meet.”*—Small Whiskey retailer quoted by **News24** **(25** **August** **2020)**

*“We are responsible for more than 300 families. These families include elderly, current workers and their children along with farm managers, winemakers, accountants, drivers and administrative staff”*—Winery owner quoted by **News24** **(25** **August** **2020)**

In addition, alarmist and emotive language in presenting the impacts of the alcohol ban on the industry were used with the use of words like “kill”, “bloodbath”, “poverty”, and “decimate”.

*South Africa’s wine producers and liquor traders have slammed the government’s extension of the ban on the sale of alcohol, saying it will kill businesses and livelihoods*—**News24** **(12 January 2021)**

*“Firstly, the previous two alcohol bans had a devastating impact on the beer industry, with 7400 job losses, R14.2 billion in lost sales revenue and 30% of breweries being forced to shut their doors”*—Alcohol industry Traders association quoted by **News24** **(28** **December** **2020)**

*“What it is doing and will do, is destroy the livelihoods of many small businesses, their employees and families, and push many people closer to extreme poverty.”*—Alcohol industry traders’ association quoted by **News** **24** **(28 July 2020)**

Another dominant strategy that was used by the alcohol industry was lobbying. This was conducted by sending letters requesting meetings with the President and government ministries responsible for the alcohol sector, such as the Ministry of Agriculture. Lobbying efforts primarily presented economic arguments against the bans, requested financial relief funds from the government, presented opposing evidence against the lockdowns, argued against the effectiveness of the ban, and proposed alternative strategies.

*“The industry is continuing with conversations with government through bodies such as the National Economic Development and Labour Council, but a solution needs to be reached soon”*—industry group quoted by **News24** **(4 August 2020)**

*In a letter sent to President Cyril Ramaphosa by the liquor traders, also on Monday, the group asked for Unemployed Insurance Fund (UIF) Temporary Employee/Employer Relief (Ters) support for the traders, financial relief in the form of a R20 000 package and a moratorium on all liquor licence fee renewals including distribution licence*—**News24** **(25** **January** **2021)**

*In a submission to the government, the Liquor Traders Association of South Africa (LTASA) proposes opening up the allowed trading hours for bottle stores, which in the draft strategy are limited to Monday, Tuesday, and Wednesday mornings until noon only*—**News24** **(16** **July** **2020)**

*“We are in consultation with the Trade and Industry department to come up with proposals on how we can potentially go back to trading in a safe environment and commitments we are making in dealing with inappropriate use of alcohol”*—Alcohol industry multinational corporation quoted by **News24** **(7 August 2020)**

The lobbying effort by industry appeared unified and networked. The alcohol industry made submissions to the government both as single business associations or trade councils and jointly. They also coordinated opposition to evidence on the effectiveness of the ban on health outcomes. The networks often included the whole alcohol conglomerate and actors from the whole supply chain (growers, manufacturers, distributors, and retailers)—these included multinational corporations like SAB, liquor traders’ associations, glass bottle makers, farmers, supermarkets, and small bars or taverns. In some media, different industry actors often referred to and reiterated arguments that had been made by other groups within the industry.

*The alcohol industry coalition commissioned Kantar—a data, insights and consulting company—to do a review of the report by Parry and others*—**News24** **(3 August 2020)**

Another strategy that was frequently used by the industry was litigation. Across all three bans, there were threats and actual court cases against the government to lift the ban. The court cases were predominantly led by SAB.

*Pressure is mounting on President Cyril Ramaphosa to lift the ban on the sale of liquor amid the nationwide lockdown—or face a court challenge.*—**SABC** **News** **(16** **April** **2020)**

*The Gauteng Liquor Forum has given Ramaphosa until Tuesday to make a decision. The forum says the lockdown regulations breach the constitutional rights of their members to freely choose their trade, occupation or profession*—**SABC** **News** **(14** **April** **2020)**

*The Southern African Agri Initiative is taking government to court today. It wants the courts to force government to lift the ban on wine sales at restaurants. They’re bringing the action on behalf of some 1hundred-and-20 wine farmers*—**News** **24** **(29 July 2020)**

*The wine producers body Vinpro is set to challenge the current lockdown ban on alcohol sales in court*—**News24** **(27 January 2021)**

*Subsequently, the country’s biggest beer brewer, AB InBev-owned SA Breweries (SAB) lodged an urgent application at the Western Cape High Court challenging the constitutionality of the ban. Industry players have also warned the ban would just fuel illicit liquor trade, which would come at the expense of taxes*—**News24** **(11 January 2021)**

*Restaurants to take on government in court over booze sales ban*—**News24** **(2** **February** **2021)**

Another strategy that was used by industry to oppose the ban was the proposition of alternatives to the alcohol ban. Common policy alternatives included de-regulation, which entailed the government using more informational techniques rather than command-and-control approaches. The industry also proposed self-regulation approaches, which involved industry actors implementing their own initiatives to target “responsible drinking”. In proposing these alternatives, the alcohol industry acknowledged the social ills that were caused by alcohol and presented themselves as good corporate citizens and part of the solution.

*He called on the government to rely “more on persuasion and cooperation, and less on control” and for a focus on “critical reinvigoration, not over-regulation”*—**News24** **(4** **August** **2020)**

*“The industry had already put measures in place to reduce the spread of Covid-19, such as withdrawing its support for entertainment events, deploying hotspot patrollers and investing R150 million to deal with binge drinking, drinking and driving, as well as underage drinking and gender-based violence”*—Alcohol industry multinational corporation quoted by **News24** **(15** **December** **2020)**

*“The industry was playing its part to address societal issues that are blamed on excessive alcohol consumption, including gender-based violence (GBV) and had started an initiative called Tavern Dialogues on Gender Based Violence”*—Alcohol industry traders association quoted by **News24** **(16** **January** **2021)**

The policy alternative proposed differed depending on whether alcohol sales were restricted or prohibited. For example, when alcohol was completely banned, the industry negotiated for regulated sales with curfews by proposing offsite consumption and reduced sale hours.

*Alcohol producers and traders have called for the restrictions to be eased and for trading to be allowed for off-site consumption*—**News24** **(12** **January** **2021)**

*“If they were to buy mixed booze, the plan would limit customers to five items, where one item is a tray or crate of beer, a box of wine, or a bottle of spirits or liqueur”*—Alcohol Industry traders association quoted by **News24** **(14** **May** **2021)**

There were minimal references to health in the arguments industry presented against alcohol prohibition. First, they noted that they agreed with the government on the need to protect the health system by reducing trauma cases. However, these statements were often followed by an emphasis on the economic impact of the ban. Health was also mentioned in the context of illicit trade and fake alcohol that might have adverse effects on consumers if alcohol was banned.

*“The fake alcohol is produced on an industrial scale and sold on the black market. It’s not safe and can cause health problems”*—Alcohol industry brands association quoted by **SABC** **News** **(17** **January** **2021)**

*“Generally, the cases are similar, they tend to find cases of pure alcohol which is something like 96% and bottles of branded products were also found which then would have been retailed with whatever processing that goes through and be sold in the broader area in KwaZulu-Natal. This is very concerning although the cases are the same, they differ in terms of scale, our concern is the risk this poses in the health of the general public.”*—Alcohol industry brands association quoted by **SABC** **News** **(16** **April** **2020)**

### 3.3. Academics, Research Organisations and Public Health Advocates

The voice of public health advocates and academics was also present in the conversations about alcohol. The most dominant voice is that of the South African Medical Council (SAMRC). They are critical of the alcohol industry and its actions. The SAMRC primarily uses evidence to demonstrate the effectiveness of the alcohol bans on trauma cases.

*“Professor Charles Parry of the South African Medical Research Council (SAMRC) has hit back at the alcohol industry following a review they commissioned on a report which informed government’s ban on the sale of liquor”*—**News24** **(3** **August** **2020)**

While the SAMRC presents evidence that demonstrates the positive impacts of the alcohol bans and restrictions, they argue that the prohibitions are not sustainable and the government should look into a more sustainable approach.

*“It is well known that from around mid-May we have been promoting a basket of regulatory measures focusing on availability, drink driving, alcohol marketing and treatment to be considered at this time in the lockdown to reduce the risk of alcohol-related trauma admissions once the first ban on alcohol sales was lifted”*—Professor Charles Parry from the SAMRC quoted by **News24** **(3** **August** **2020)**

### 3.4. Non-Governmental Organisations (NGOs)

There were a few NGOs whose views emerged in the media in relation to alcohol bans, these were primarily welfare NGOs that had responsibility for women and children, alcohol rehabilitation organizations, and one association that lobbied against the criminalization of drugs. While none of these organizations were reported to lobby for or against the regulations, their views were presented in the media. The alcohol rehabilitation organizations and the association against the criminalization of drugs argued for a balanced view of the impact of alcohol and prohibition.

*Organisations that deal with alcohol-related problems have warned that the ban may have negative consequences for alcoholics. Head of addiction treatment at Crossroads Recovery Centre, says the abrupt withdrawal of alcohol could result in a loss of consciousness or seizures for those who are addicted to it*—**SABC** **News** **(14** **July** **2020)**

*The chairperson of the South Africa Drug Policy Initiative, Doctor Keith Scott has warned that alcohol abuse poses other dangers apart from causing an increase in trauma cases that put pressure on hospitals’ casualty wards*—**News24** **(2** **February** **2021)**

Welfare organizations for children and women supported the bans citing that alcohol exacerbated the incidences of domestic abuse to women and children.

*Director of the Phoenix Child Welfare– says alcohol is a contributing factor in up to 80 percent of all cases of Gender-Based Violence*—**SABC** **News** **(14** **April** **2020)**

*“Our stance was that it should not be lifted; however, we support the president. Remember when someone is under the influence of liquor, there is a change in behavior and alcohol-related arguments. Most of the perpetrators commit offences when they are under the influence of alcohol”*—Gender based violence NGO quoted by **SABC** **News** **(20** **August** **2020)**

## 4. Discussion

Our analysis shows that COVID-19 presented a critical juncture for alcohol-related policy changes in South Africa. However, whether this results in sustainable policy changes in alcohol regulation will depend on a variety of factors. In line with Shiffman & Smith’s determinants of political priority for policy change, we explored several factors which included the power of actors involved, how they framed the policy issue, political contexts and the nature of the issue, such as evidence on the need for or effectiveness of a policy solution [11].

### 4.1. Actors Involved and Strategies Employed

The government, the opposition, industry, academics and public health advocates, and NGOs employed different strategies to support or oppose the ban on alcohol sales during the COVID-19 pandemic. The media not only reported on these strategies but the actors themselves used the media as a strategic outlet to support or oppose the ban by disseminating information and framing the issue in different ways to justify their position (for or against). Similar observations were found in a study that examined the politics of alcohol policies presented in South African media [9]. Additionally, the media itself can be seen as an important actor by the way different networks choose to report news stories. For instance, industry views were predominantly presented on News24 with comparatively little representation from government and little to no representation from NGOs, meaning viewers of that network were dominated by the industry narrative without alternative viewpoints. In general, public health advocates and NGOs featured infrequently in the media, which may present issues with retaining the policy (or similar alcohol controls) in the future. While the public health NGO voice may be limited, the SAMRC was well presented in the media. The SAMRC played a crucial role in countering industry evidence and presented a nuanced view that suggests alcohol prohibitions as a temporary measure and regulation of access and supply of alcohol as a more sustainable approach. Media advocacy is a well-documented and successful strategy for influencing health policy [22]. Having balanced voices in the media has the potential to move the policy discussion forward. Media narratives can become normative beliefs and evidence from Australia’s nightlife policies (commonly referred to as ‘last drinks’ or ‘lockout’ laws) provide an example of how policies can be removed when industry narratives are consistently presented in the media and go unchallenged by health groups [23]. The ability to retain South Africa’s new policies may be threatened by the lack of health representation in the media.

Collaboration and consistent messaging were important strategies identified in our analysis. The introduction of the COVID-19 related alcohol bans through an inter-ministerial structure of the National Coronavirus Command Council brought a unique opportunity for collaboration and created a window for policy change [14]. However, there was different messaging coming from different sectors of government and they lacked cohesion. These findings are congruent with another study which found that different government departments advance their own interests and objectives during alcohol policy formulation in South Africa [24]. Networks and cohesion amplify the messaging of policy actors [14,25], this is lacking in pro-ban groups and government actors and thus, it may present another difficulty in retaining the policy momentum gained from COVID-19 alcohol-related bans.

In contrast, the much stronger opposing industry voice was more cohesive and supported by the opposition party. The industry actors were networked and employed a wide range of approaches including lobbying various political parties and representatives, and litigation against the government, while the government actors typically took on a defensive stance. The whole alcohol industry was comparatively much more united with consistent messaging and representation from many industry actors across the supply chain, including growers, glass bottle makers, boutique bars and informal taverns or shebeens. This is unlike previous debates on alcohol regulation in South Africa where there has been a divide between licensed traders in upper-income sectors and unlicensed or less formalized traders that are often found in low-income communities [9]. This shows a change in industry tactics and a deliberate intention to collaborate and align industry actors. Within these networks, industry actors lobbied various political parties and representatives and litigated government as individual sectors and as networks. These are common and effective strategies used by industry groups, including the tobacco and food industries, to avoid or delay government regulation [26,27].

### 4.2. Political Context and Issue Framing

The context of COVID-19 and the existing high rates of alcohol harm was important for framing the issue and demonstrating a need for comprehensive alcohol controls. When introducing the ban, the government primarily framed the restrictions as necessary to reduce the burden on hospitals during the pandemic by lowering the number of alcohol-related trauma cases presenting to emergency rooms. However, different ministries within government (transport, police, and social services) appeared in the media, framing the ban as important for reducing a range of other alcohol-related harm including street violence, road accidents, alcohol abuse and dependency, and family and domestic violence. Alcohol has predominantly been presented in South African media in terms of acute problems, such as interpersonal violence, which frame the issue in terms of social risks more so than health risks [28,29]. While COVID-19 related pressures on health care systems were common across the globe, South Africa had to anticipate overburdening of the health care system from both COVID-19 cases as well as alcohol-related trauma cases that already overburden the health system in a non-pandemic context [5,29]. This indicates the gravity of alcohol-related harm in the country. The reliance on the ban as a mechanism for addressing a range of issues across the ministries indicates governance failures in implementing and enforcing laws, such as crime prevention, road safety, and reduction of alcohol consumption. Challenges with law enforcement related to traffic law enforcement and crime prevention in South Africa are well documented [28,30,31]. These failures in the existing regulatory environment, and the current policy momentum created by COVID-19, could present an opportunity to retain components of the new laws and/or introduce additional policies to improve the existing regulatory environment. If the various government departments align their objectives and health groups and NGOs use more coordinated and consistent messaging in the media, this may prove particularly effective.

The economic impact of the COVID-19 alcohol ban was the most prominent argument against regulation. This argument was predominantly made by all sectors of the alcohol industry and by the leading opposition parties. The Ministry of Finance also used this frame, acknowledging the economic impact of the ban, but interestingly they did not present the alternative framing of other departments, in terms of decreased hospitalizations and saved costs to the health system. This once more highlights some of the incoherence within government. Framing is used to present arguments in ways that appeal to certain values. For example, there were numerous references to vulnerable small businesses and farmworkers. Framing policies as harmful to vulnerable populations is a commonly used tactic by international corporations for example in relation to sugar taxes and tobacco control policies [32,33]. Interestingly, proponents of the ban also used vulnerability framing but in relation to the vulnerability of health systems. This may reflect an acknowledgment of already well-known health system challenges. Both opponents and proponents of the ban viewed alcohol consumption as an individual or lifestyle issue. In the periods when alcohol has not been banned but restrictions remain in place, individual responsibility has been emphasized by both industry and government actors. The emphasis on individual responsibility is part of the neoliberal framing that is preferred by industry to evade government regulation [34,35]. The government may also be using this frame to soften some of the negative media surrounding economic impacts and to demonstrate support for the industry when they can. However, if alcohol regulation is to improve in South Africa over the long term, supporters should be wary of this framing and how it inevitably plays out to the detriment of population health goals. From a public health perspective, this type of framing, also known as ‘lifestyle drift’ is an ongoing barrier to action on the social determinants of health. This is because even when problems are initially described in terms of a need for an upstream or high-level policy approach, they invariably shift to a downstream approach and thus focus largely on individual lifestyle factors [36,37]. Government agencies focused on health promotion often rely upon reductionist approaches that when rolled out are both simplistic and paternalistic; often focusing solely on public education to motivate behavior change, thus perpetuating an individual-focused narrative [38].

### 4.3. Use of Evidence and Policy Alternatives

Evidence was fundamental to the adoption and implementation of the alcohol ban and will remain vital to ensuring the acceptance and retention of certain alcohol controls into the future. Restricting the availability of alcohol through temporal controls, including bans and other time-based restrictions, are ‘best buys’ for the reduction of alcohol consumption and prevention of alcohol harm [39,40,41]. This evidence was cited in the media by supporters of the bans. Additionally, supporters used new evidence as it emerged, thus demonstrating the real-time positive impact of the ban on reducing trauma-related hospitalizations [42,43]. In contrast, opponents of the ban presented opposing evidence against the ban and the COVID-19 lockdowns, arguing against their effectiveness and proposing alternative strategies. Evidence against the effectiveness of the ban was not cited but the narrative of “the ban being ineffective” was frequently reported in the media. Opponents did, however, present evidence demonstrating the negative economic impact, for instance, citing large profit losses.

In proposing alternative strategies, they took two different approaches. When a total ban was in place, they argued for time-based restrictions, but when time-based restrictions were in place, they argued for self-regulation whereby the alcohol industry implements and monitors the alcohol controls. This demonstrates that the alcohol industry will always support de-regulation and argue against even modest controls. Another proposed alternative was public-private partnerships which entailed alcohol retailers partnering with the government to reduce overconsumption through corporate social responsibility initiatives. When the 2013 draft Control of Marketing of Alcoholic Beverages Bill (which sought to ban alcohol advertising) was introduced, the alcohol industry lobbied for self-regulation, enforcement of existing laws, and increased their corporate social responsibility activities. In addition, the industry introduced public-private partnerships and self-regulatory initiatives as counter-strategies for the alcohol ban [24]. They also suggested the ban increased illicit trade which is not supported by evidence and is another common strategy used by the tobacco industry to counter regulations [44,45]. This again demonstrates the consistency of industry messaging, using the same narratives to prevent any type of alcohol regulation, COVID-19 related or otherwise. Evidence of the impacts of the alcohol ban on health is rarely mentioned by industry except in relation to the harm that may be caused by homemade alcohol.

## 5. Conclusions

Overall, these findings indicate that the alcohol industry is consistent in its use of strategies to counter alcohol regulations, both in the case of COVID-19 and with respect to past regulatory actions. The reliance on bans by each of the government ministries, as well as concerns from actors, such as NGOs, indicates a failure in the existing regulatory environment. This demonstrates a need for evidence-based alcohol regulation to remain long-term, post the COVID-19 pandemic. This would, however, represent a paradigm shift within the system, something that is both difficult to achieve and maintain [46]. Ensuring that the right kinds of narratives around health issues like alcohol-related social and health harm in South Africa are constructed and promulgated in a cohesive manner will be critical for ensuring that long-term and sustainable change occurs [47]. Despite the absence of the community and pro-public health voice, or coordinated action from them, we believe that the COVID-19 alcohol bans and restrictions still provided an opportunity to debate and demonstrate the necessity of a comprehensive policy to regulate alcohol.

## 6. Study Limitations

This study presents important findings on how different actors navigated the COVID-19 related alcohol bans and what these strategies might mean for future regulation of alcohol in South Africa. Notwithstanding this contribution, this study was subject to some limitations. First, the strategies presented here were based on the research team’s interpretation of what was presented in the mainstream media. While this analysis captures the voices of dominant players, those who were not presented within mainstream media may have been excluded. There may also be bias within media representation, choosing to present certain actors’ viewpoints over others. Future research could fill this potential gap in knowledge through the collection of in-depth interview data with different types of actors. Second, our analysis was limited to the first three alcohol bans; since these bans, there have been further prohibitions and restrictions and accordingly, the responses by different types of actors may have changed. Further monitoring of these responses and strategies will be crucial for advancing regulation studies and alcohol policy. Third, our analysis was limited to two media houses SABC News and News24, therefore, while this paper gives a good snapshot of the actors, how they are involved and their strategies used to influence regulation, some actors ‘perspectives may have been omitted.

## Data Availability

Data can be made available upon request via email to the authors.
